# Future directions in neuro-optometry

**DOI:** 10.2217/cnc-2020-0013

**Published:** 2020-09-24

**Authors:** Kenneth J Ciuffreda, Barry Tannen

**Affiliations:** 1 Department of Vision Sciences, SUNY College of Optometry, New York City, NY 10036, USA

**Keywords:** brain injury, neuro-optometry, optometry, public health, vision care

The area of neuro-optometry has evolved over the past 40 years to provide essential vision care for the brain-injured population. Here we present some proposed future directions in the field that could improve and expand this area of patient care.

What do we mean by neuro-optometry? It represents a broad and evolving subspecialty within the profession of optometry [1,2]. More specifically, neuro-optometry deals with the diagnosis and treatment of vision-based problems commonly found in the brain-injured population (e.g., concussion and cerebrovascular accident). It “*addresses the oculomotor, accommodative, visuomotor, binocular, vestibular, perceptual/visual information processing and specific ocular/neurobiological sequalae of this population”* [2] (see also the COVD and NORA websites). Some of these problems include marked light and visual motion sensitivity, vergence dysfunction, oculomotor-based reading problems, blur, multimodal sensory integration deficits and deficient visuomotor planning and execution. Treatment includes vision therapy, lenses, prisms, selective occlusion and lens tints/coatings. The aforementioned neuro-optometric rehabilitation follows the scientifically based tenets of neuroplasticity, incorporating the principles of perceptual and motor learning [2]. At last, it is remarkable that the vision problems in many of these patients can be remediated, at least in part, even in an older damaged brain.

Where is the exciting field of neuro-optometry headed in the future? This is an important question as our crucial role in traumatic brain injury, and more broadly the diagnostic categories of acquired brain injury, as well as basic neurological/developmental disorders continues to expand. This includes both diagnostic and therapeutic aspects of vision care. It is, and will continue, to be performed in conjunction with other healthcare providers, as needed (e.g., the physiatrist, cognitive psychologist, occupational therapist, vision therapist), as well as in partnership with industry and the university (e.g., for development of specialized computer hardware/software and test devices).

There are several possible areas of future focus and expansion of vision care. Some of these include:

- Development and use of precision chromatic tints, very narrow-spectrum filters and custom chromatic digital device software for treatment of the common condition of photosensitivity [3,4], as well as the rarer visual snow syndrome [5]. These chromo-optical tools may also help many patients with visual motion sensitivity by reducing the luminance and/or color saturation of the offensive visual stimuli in the environment [6];
- Development and use of variable/adjustable prismatic spectacles for daily patient monitoring, and immediate relief from/assistance for inconsistent and large near phoria magnitude and the resultant transient diplopia. These spectacles could also be designed to provide continuous vergence therapy during one’s daily activities. In addition, a simple and compact, video-based eye movement monitoring system could be incorporated that produces a tonal signal when fusion is disrupted, thus providing an auditory cue to regain binocularity [7];
- Development and use of virtual/augmented reality constructs to evaluate and treat common perceptual dysfunctions found in these populations [8]. Some deficits include abnormal egocentric localization (i.e., the sense of ‘straight-ahead’), abnormal sense of visual vertical and increased visual motion sensitivity. Work has already begun related to the aforementioned diagnoses using a clinically based egocentric test device [9], a rod-and-frame apparatus using a virtual reality construct [10] and a full-field visual motion habituation system [6], respectively. However, more work is warranted with emphasis on direct clinical application;
-  Development and use of more sophisticated oculomotor testing and training programs incorporating a full range of conditions and platforms. Some examples include use in a conventional computer system, in a controlled virtual/augmented reality setting and in an active, free-space, visuo-motor-based environment to simulate naturalistic conditions. One current, computer-based software training system incorporates many useful and novel aspects [11]. Along with conventional fixation, saccade, pursuit, vergence, vestibular and accommodative training stimuli, the system’s software has the ability to introduce visual and auditory distractors to ‘load’ the task, as well as the incorporation of the rapid serial visual presentation paradigm to control and modulate visual attention [12]. At last, oculomotor-based, auditory feedback could be introduced to enhance motor learning and thus shorten the training timeframe [7];
- Development and use of both dynamic posturography [13] and dynamic gait analysis [14] to assess baseline balance and ambulation, respectively, to test the effect of a therapeutic intervention (e.g., vision therapy, yoked prisms) and to monitor for any natural recovery or regression over time. At last, one might incorporate auditory feedback information correlated with overall gait dynamics and gait rhythmicity. That is, a continuous, pitch-modulated, auditory signal related to changes in leg trajectory (e.g., position, velocity) would be presented. Thus, the patient would both see and ‘hear’ themselves moving in visual space. Presence of such dual-mode information would likely result in faster recovery of deficient, visually guided, ambulatory-based activities. This technique could also be used to assess and train steady-state balance;
- One could incorporate auditory feedback information to train the frequently abnormal accommodative system [15]. A continuous, pitch-modulated signal related to either steady-state accuracy or dynamic changes in dioptric trajectory would be presented. Thus, the patient would both see visually based blur changes and ‘hear’ themselves focus on a target in visual space. This technique could also be employed with the vergence eye movement system used to track objects in depth. In this latter case, there would be the transient, visually based diplopic changes with the correlated tonal information continually present;
- One could also employ visual and/or auditory feedback information to assess and train the speed and accuracy of visuomotor activities, such as grasping for a small object (e.g., a pin). For example, one could use an electronic maze tracing device [16]. Here, the patient manually controls an ‘electronic’ pen to trace within a complicated maze as rapidly and accurately as possible. Each time the pen deviates from the path, a tone is generated. These accumulated errors are automatically counted, as well as the time for completion. This could also be performed in a virtual reality setting.

These suggestions represent just a handful of possible future directions. The contemporary neuro-optometrist is currently performing well in the testing and training of the brain-injured population with their constellation of visual dysfunctions [17–19]. However, with a more expansive and futuristic ‘vision’ of the range of possibilities, in conjunction with an evolving and global conceptual model of vision care [19], it is likely that our capabilities and performance can be accelerated to attain even higher levels of patient gratification and professional success.
